# Supplementary material for: SCRaMbLE: A Study of Its Robustness and Challenges through Enhancement of Hygromycin B Resistance in a Semi-Synthetic Yeast
Source: Bioengineering (Basel). 2021 Mar 23;8(3):42. doi: 10.3390/bioengineering8030042 (PMC8004914; doi:10.3390/bioengineering8030042)
Supplement: Supplementary file 1 [file bioengineering-08-00042-s001.pdf]

Table 1. Structural variations detected within SCRaMbLEd strains by nanopore sequencing.

Structural variation	start	stop	Length (bp)	ORF(s) involved	Note
**YCy1188**					
INS-0	709313	710469	1131	*YBR265W*	Insertion into chrVIII
**HYG2.1**					
DEL-1	422613	427123	4509	*YBR107C, YBR108W*	
INS-0	709313	710432	1118	*YBR265W*	Insertion into chrVIII
**HYG2.2**					
INVDUP-2	681	80126	79445	*YBL106C, YBL105C, YBL104C, YBL103C, YBL102W, YBL101C, YBL100W-B, YBL100C, YBL099W, YBL098W, YBL097W, YBL096C, YBL095W, YBL094C, YBL093C, YBL092W, YBL091C-A, YBL091C, YBL090W, YBL089W, YBL088C, YBL087C, YBL086C, ARS202, YBL085W, YBL084C, YBL083C, YBL082C, YBL081W, YBL080C, YBL079W, YBL078C, YBL077W, YBL076C, YBL075C, YBL074C, YBL073W, YBL072C, YBL071C-B, YBL071W-A, YBL071C, YBL070C, YBL069W, YBL068W-A, YBL068W, ARS203, YBL067C*	
INV-3	639	34603	33961	*YBL106C, YBL105C, YBL104C, YBL103C, YBL102W, YBL101C, YBL100W-B, YBL100C, YBL099W, YBL098W, YBL097W, YBL096C, YBL095W, YBL094C, YBL093C, YBL092W, YBL091C-A, YBL091C, YBL090W, YBL089W*	
DEL-4	215564	216524	957	No ORF	
DEL-5	236434	237757	1324	*YBR014C*	
DUP-6	469009	483416	14406	*YBR133C, YBR134W, YBR135W, YBR136W*	
DEL-7	624704	642485	17779	*YBR216C, YBR217W, YBR218C, YBR219C, YBR220C, YBR221C, YBR221W-A, YBR222C, YBR223C, YBR224W, YBR225W, YBR226C*	
INV-8	710532	717598	7066	*YBR270C, YBR271W, YBR272C*	
INS-0	709308	710434	1122	*YBR265W*	Insertion into chrVIII
**HYG2.4**					
DEL-9	214641	216499	1853	*YBL001C, ARS208, CEN2*	
INV-10	307288	320620	13344	*YBR050C, YBR051W, YBR052C, YBR053C, YBR054W, YBR055C, YBR056W*	
DEL-11	375801	389571	13767	*YBR082C, ARS214, YBR083W, YBR084W, YBR084C-A, YBR085W, ARS215, YBR085C-A*	
INV-12	620275	627528	7219	*YBR214W, YBR215W, YBR216C, YBR217W*	
INS-0	709312	710432	1116	*YBR265W*	Insertion into chrVIII

Table 2. Yeast strains used and generated in this study. Genotypes of the SCRaMbLEd strains (HYG2.1 to HYG2.5) are exclusive of the structural variations caused by SCRaMbLE.

Strain ID	Alternative name	Genotype	Reference
YCy1188	synIIv1.4, synII-*YBR265W-URA3*, YSy114	*MATa his3Δ1 leu2Δ0 LYS2 met15Δ0 ura3Δ0 synII::URA3*	7
YCy1189	synIIv1.3, YSy033	*MATa his3Δ1 leu2Δ0 LYS2 met15Δ0 ura3Δ0 synII*	7
YCy2915	BY4742	*MATα his3Δ1 leu2Δ0 lys2Δ0 ura3Δ0*	46-47
YCy2917	BY4742-pRS413	*MATα his3Δ1 leu2Δ0 lys2Δ0 ura3Δ0 [HIS3 pRS413]*	This study
YCy2919	synII-pRS413	*MATa his3Δ1 leu2Δ0 LYS2 met15Δ0 ura3Δ0 synII::URA3 [HIS3 pRS413]*	This study
YCy2918	synII-pSCW11-Cre-EBD	*MATa his3Δ1 leu2Δ0 LYS2 met15Δ0 ura3Δ0 synII::URA3 [HIS3 pSCW11-Cre-EBD]*	This study
YCy2983	*YBR101C (fes1)Δ*BY4742	*MATα his3Δ1 leu2Δ0 lys2Δ0 ura3Δ0 fes1Δ::kanMX*	42
YCy2984	*YBR132C (agp2)Δ*BY4742	*MATα his3Δ1 leu2Δ0 lys2Δ0 ura3Δ0 agp2Δ::kanMX*	42
YCy4021	HYG2.1	*MATa his3Δ1 leu2Δ0 LYS2 met15Δ0 ura3Δ0 synII::URA3*	This study
YCy4022	HYG2.2	*MATa his3Δ1 leu2Δ0 LYS2 met15Δ0 ura3Δ0 synII::URA3*	This study
YCy4023	HYG2.3	*MATa his3Δ1 leu2Δ0 LYS2 met15Δ0 ura3Δ0 synII::URA3*	This study
YCy4024	HYG2.4	*MATa his3Δ1 leu2Δ0 LYS2 met15Δ0 ura3Δ0 synII::URA3*	This study
YCy4025	HYG2.5	*MATa his3Δ1 leu2Δ0 LYS2 met15Δ0 ura3Δ0 synII::URA3*	This study
YCy4132	*YBR107C(iml3)Δ* BY4742	*MATα his3Δ1 leu2Δ0 lys2Δ0 ura3Δ0 iml3Δ::kanMX*	42
YCy4133	*YBR108W (aim3) Δ*BY4742	*MATα his3Δ1 leu2Δ0 lys2Δ0 ura3Δ0 aim3Δ::kanMX*	42

Strain ID	Alternative name	Genotype	Reference
YCy4150	*YBR014C⊿*BY4742	*MATα his3Δ1 leu2Δ0 lys2Δ0 ura3Δ0,YBR014C⊿::KanMX*	42
YCy4151	*YBR216C⊿*BY4741	*MATa his3Δ1 leu2Δ0 met15Δ0 ura3Δ0,YBR216C⊿::KanMX*	42
YCy4152	*YBR217W⊿*BY4741	*MATa his3Δ1 leu2Δ0 met15Δ0 ura3Δ0, YBR217W⊿::KanMX*	42
YCy4153	*YBR218C⊿*BY4741	*MATa his3Δ1 leu2Δ0 met15Δ0 ura3Δ0, YBR218C⊿::KanMX*	42
YCy4154	*YBR219C⊿*BY4741	*MATa his3Δ1 leu2Δ0 met15Δ0 ura3Δ0, YBR219C⊿::KanMX*	42
YCy4155	*YBR220C⊿*BY4741	*MATa his3Δ1 leu2Δ0 met15Δ0 ura3Δ0, YBR220C⊿::KanMX*	42
YCy4156	*YBR221C⊿*BY4741	*MATa his3Δ1 leu2Δ0 met15Δ0 ura3Δ0, YBR221C⊿::KanMX*	42
YCy4157	*YBR221W-A⊿*BY4742	*MATα his3Δ1 leu2Δ0 lys2Δ0 ura3Δ0, YBR221W-A⊿::KanMx*	42
YCy4158	*YBR222C⊿*BY4742	*MATα his3Δ1 leu2Δ0 lys2Δ0 ura3Δ0,YBR222C⊿::KanMX*	42
YCy4159	*YBR223C⊿*BY4742	*MATα his3Δ1 leu2Δ0 lys2Δ0 ura3Δ0,YBR223C⊿::KanMX*	42
YCy4160	*YBR224W⊿*BY4742	*MATα his3Δ1 leu2Δ0 lys2Δ0 ura3Δ0, YBR224W⊿::KanMX*	42
YCy4161	*YBR225W⊿*BY4742	*MATα his3Δ1 leu2Δ0 lys2Δ0 ura3Δ0, YBR225W⊿::KanMX*	42
YCy4162	*YBR226C⊿*BY4742	*MATα his3Δ1 leu2Δ0 lys2Δ0 ura3Δ0,YBR226C⊿::KanMX*	42
YCy4163	*YBL001C⊿*BY4742	*MATα his3Δ1 leu2Δ0 lys2Δ0 ura3Δ0,YBL001C⊿::KanMX*	42
YCy4164	*YBR082C⊿*BY4741	*MATa his3Δ1 leu2Δ0 met15Δ0 ura3Δ0, YBR082C⊿::KanMX*	42
YCy4165	*YBR083W⊿*BY4742	*MATα his3Δ1 leu2Δ0 lys2Δ0 ura3Δ0, YBR083W⊿::KanMX*	42
YCy4166	*YBR084W⊿*BY4742	*MATα his3Δ1 leu2Δ0 lys2Δ0 ura3Δ0, YBR084W⊿::KanMX*	42
YCy4167	*YBR084C-A⊿*BY4742	*MATα his3Δ1 leu2Δ0 lys2Δ0 ura3Δ0, YBR084C-A⊿::KanMX*	42
YCy4168	*YBR085W⊿*BY4742	*MATα his3Δ1 leu2Δ0 lys2Δ0 ura3Δ0, YBR085W⊿::KanMX*	42
YCy4169	*YBR085C-A⊿*BY4742	*MATα his3Δ1 leu2Δ0 lys2Δ0 ura3Δ0, YBR085C-A⊿::KanMX*	42

Supplementary Fig1. Ong et al, 2020

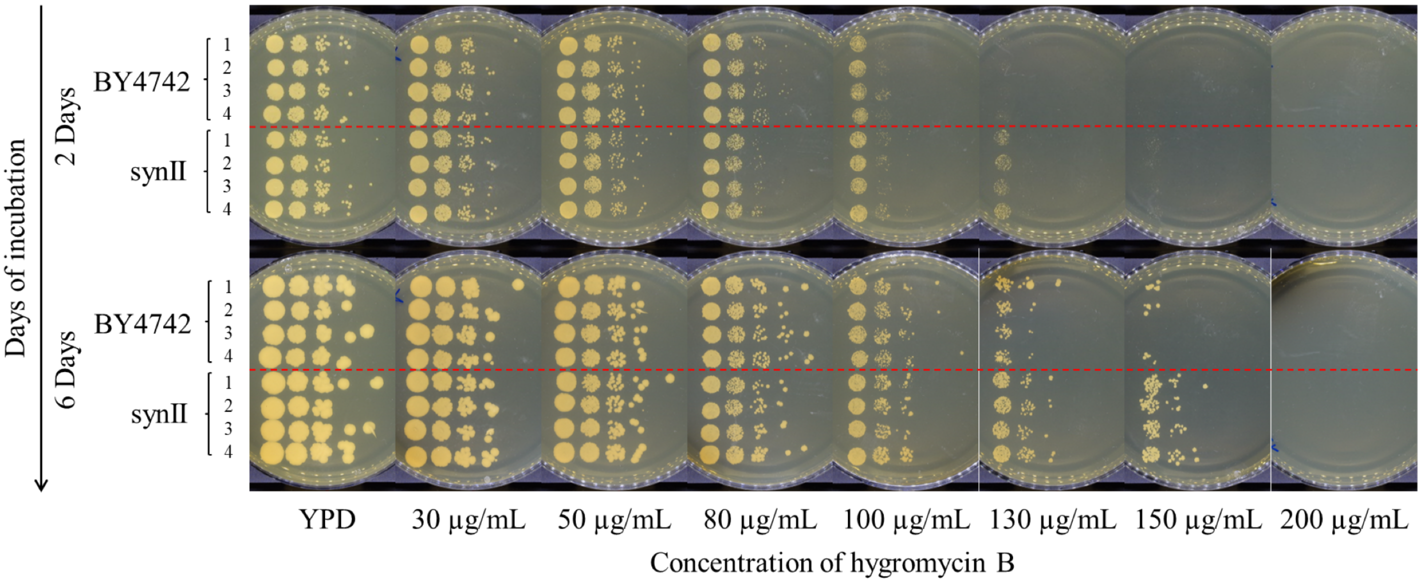

Figure S1. The parental synII strain (YCy1188) and the wild type strain (BY4742) were benchmarked for their resistance towards hygromycin B. The lethal concentration of hygromycin B was identified to be 200 µg/mL. Number 1 to 4 indicate independent biological replicates.

Supplementary Fig2. Ong et al, 2020

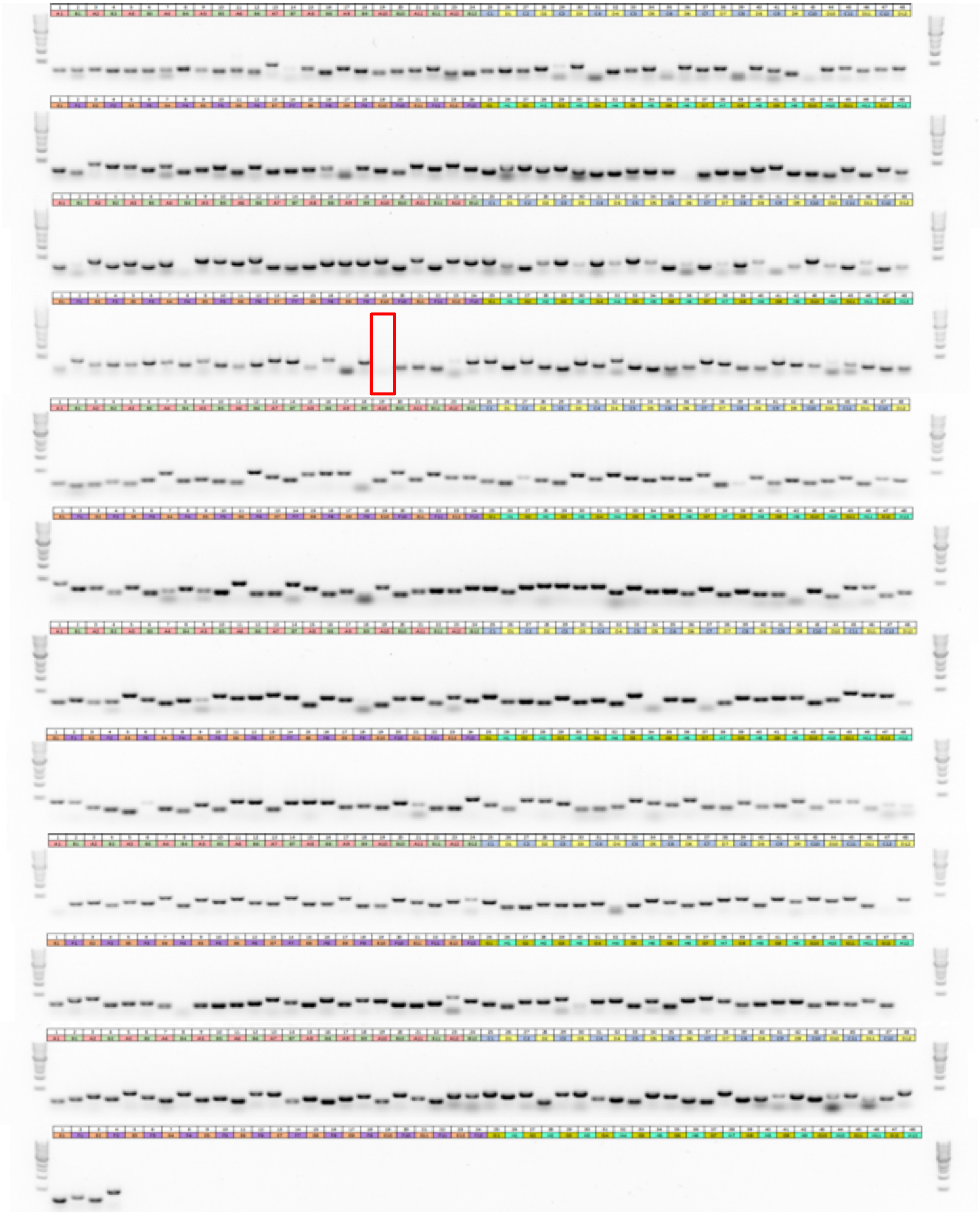

Figure S2. PCRTag analysis of HYG2.2 to identify gene deletions after SCRaMbLE. Red box indicated the absence of amplicon for *GRX7* (*YBR014C*). PCRTag analysis of HYG2.1, HYG2.3, HYG2.4 and HYG2.5 were performed similarly.

Supplementary Fig3. Ong et al, 2020

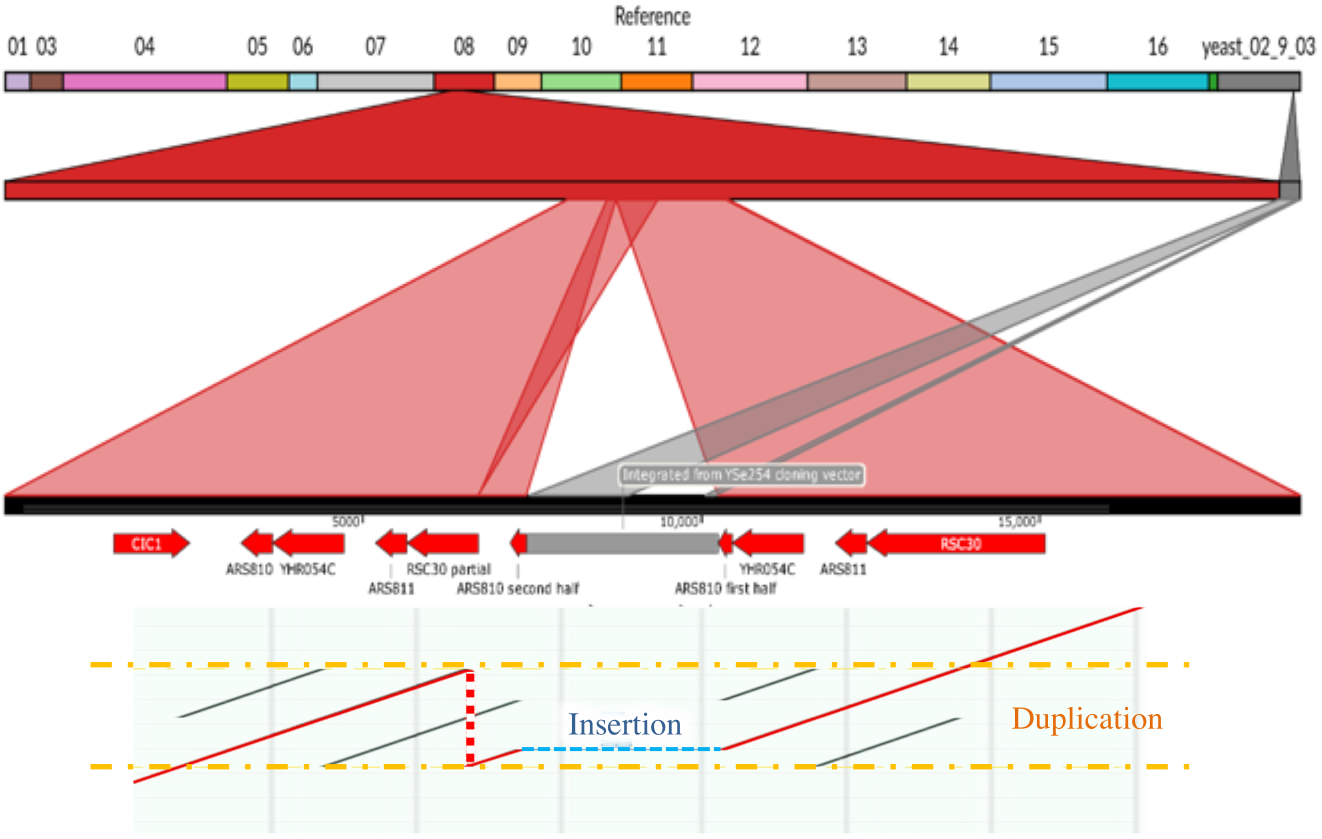

Figure S3. Sequencing data identified that the synthetic recoded *TSC10* on synII has not been replaced but the wildtype *TSC10* is present as additional copy on chromosome 8.
